# Dipyanone, a new methadone-like synthetic opioid: In vitro and in vivo human metabolism and pharmacological profiling

**DOI:** 10.1007/s00204-025-04023-1

**Published:** 2025-04-29

**Authors:** Diletta Berardinelli, Johannes Kutzler, Omayema Taoussi, Simona Zaami, Simona Pichini, Giuseppe Basile, Francesco Paolo Busardò, Volker Auwärter, Jeremy Carlier

**Affiliations:** 1 Present Address: Marche Polytechnic University, Department of Biomedical Sciences and Public Health, Section of Legal Medicine, Unit of Forensic Toxicology, Ancona, Italy; 2 Institute of Forensic Medicine, Forensic Toxicology, Medical Center - University of Freiburg, Faculty of Medicine, Freiburg, Germany; 3 Hermann Staudinger Graduate School, University of Freiburg, Freiburg, Germany; 4 Sapienza University of Rome, Department of Anatomical, Histological, Forensic and Orthopaedic Sciences, Rome, Italy; 5 National Institute of Health, National Centre On Addiction and Doping, Rome, Italy

**Keywords:** Dipyanone, New synthetic opioid, Metabolism, Pyrrolidine ring opening, Opioid receptor

## Abstract

The emergence of novel synthetic opioids (NSOs) poses significant challenges to public health, toxicologists, and law enforcement. Since the comprehensive scheduling of fentanyl analogues in many countries worldwide, several non-fentanyl NSOs have emerged on the illicit drug market, increasing the risks of addiction and fatal overdoses. Dipyanone, a methadone-like NSO, was first identified in drug seizures in 2021 and later also in postmortem cases. This study aimed to assess the metabolism of dipyanone in humans using in silico predictions, in vitro human hepatocyte incubations, and authentic urine specimens. Dipyanone was primarily metabolised through the opening of the pyrrolidine ring to form the corresponding *N*-butan-4-ol or *N*-butanoic acid compound, followed by cyclisation to 4’-[2-ethylidene-5-methyl-3,3-diphenylpyrrolidin-1-yl]butan-1’-ol (EMDPB) and 4’-[2-ethylidene-5-methyl-3,3-diphenylpyrrolidin-1-yl]butanoic acid (EMDPBA); other metabolic transformations included hydroxylation, reduction, and *O*-glucuronidation. We propose EMDPB and EMDPBA as specific biomarkers of dipyanone consumption. Additionally, dipyanone’s activation of µ- (MOR), κ- (KOR), and δ- (DOR) opioid receptors was evaluated with a homogeneous time-resolved fluorescence-based GTP G_i_ binding assay. The half-maximal effective concentration (EC_50_) and maximum effect (*E*_max_) were 96.8 nM and 106% (compared to fentanyl at MOR), 380.4 nM and 13% (compared to U-50488 at KOR), and 1067 nM and 56% (compared to SNC-80 at DOR). Dipyanone’s opioid receptor activation pattern was similar to that of methadone, with a strong agonistic effect at MOR, potentially inducing potent analgesic effects with health risks through the central nervous system and respiratory depression, as well as an abuse and dependence potential. Further pharmacokinetic and pharmacodynamic studies should be conducted to better understand the toxicological relevance of dipyanone.

**Supplementary Information:**

The online version contains supplementary material available at 10.1007/s00204-025-04023-1.

## Introduction

According to the European Drug Report 2024 from the European Union Drugs Agency (EUDA), 81 different novel synthetic opioids (NSOs) have been reported on the illicit drug market since 2009. Fentanyl analogues largely dominated the NSO market until 2019 (EUDA 2024; Pichini et al. 2017). However, following their scheduling in the United States in 2018 and in China in 2019, new opioids subclasses, such as the nitazenes, have emerged. Nitazenes have recently become a major source of concern due to their association with addiction and overdose fatalities (EUDA 2024; Montanari et al. 2022).

In 2021, two opioids structurally and pharmacologically related to methadone appeared on the illicit drug market, forming a new NSO subclass known as methadone analogues (EUDA 2024; EMCDDA 2023). Among these substances, *N*-pyrrolidino methadone [4,4-diphenyl-6-(1’-pyrrolidinyl)-3-heptanone], also named dipyanone, was first described in 1946 by Scott et al. as an analgesic drug. It was found to exhibit an activity similar to methadone in rats, dogs, and humans, with the same median lethal dose in mice (Bockmühl and Ehrhart 1949; Scott et al. 1946). In vitro experiments further demonstrated that dipyanone is roughly as active as methadone at the µ-opioid receptor, with a half-maximal effective concentration (EC_50_) of 39.9 nM and an efficacy (E_max_) of 155% compared to hydromorphone (Vandeputte et al. 2023).

Although dipyanone has a lower experimental potency compared to other popular NSOs, it may still pose considerable health risks, including central nervous system depression, respiratory depression, bradycardia, and hypotension; and it very likely shows an abuse potential similar to methadone. Although dipyanone is not explicitly mentioned in drug control laws or regulations, it may be subject to restrictions under analogue laws in various countries. Dipyanone was first detected on the illicit drug market in Germany in 2021, and a few months later in Slovenia (European Database on New Drugs 2023), highlighting the rapid distribution of new synthetic opioids across borders. In 2022 and 2023, four dipyanone-related fatalities occurred in Germany. Dipyanone concentrations ranged from 80 to 5500 ng/mL in urine and from 720 to 1400 ng/mL in blood (own unpublished data). (Vandeputte et al. 2023).

Dipyanone was also detected alongside other NSOs in the blood of a postmortem case in the USA, with a measured concentration of 370 ng/mL (Vandeputte et al. 2023).

However, data on dipyanone concentrations in biological specimens and pharmacodynamics are limited, with no information available on its pharmacokinetics. Understanding the pharmacology of dipyanone in humans is essential for determining its role in intoxications and fatalities in clinical and forensic casework. Particularly, identifying specific metabolite biomarkers of consumption is crucial for improving detection, diagnosis, and treatment outcomes.

In the present study, we aimed to investigate the metabolic fate of dipyanone in humans and identify specific metabolite biomarkers of consumption using in silico predictions, pooled human hepatocyte incubations, and analysis of biological samples. Additionally, we evaluated dipyanone’s activation of µ- (MOR), δ- (DOR), and κ- (KOR) opioid receptors using a novel homogeneous time-resolved fluorescence (HTRF®)-based GTP G_i_ binding assay to further understand the drug’s effects.

## Materials and methods

### Chemicals and reagents

Pure standards of dipyanone, fentanyl, metonitazene, methadone-d9, SNC-80, and U-4880 were purchased from Cayman Chemical (Ann Arbor, Michigan, USA), diclofenac, while ammonium acetate, β-glucuronidase and dimethyl sulfoxide (DMSO) were bought from Sigma Aldrich (Milan, Italy). The standards were prepared as 1 mg/mL solutions in methanol and stored at – 20 °C until analysis. Carlo Erba (Cornaredo, Italy) supplied LC–MS grade methanol, acetonitrile, water, and formic acid. William’s medium E, HEPES buffer (2-[4-(2-hydroxyethyl)-1-piperazinyl]ethanesulfonic acid), and *L*-glutamine were obtained from Sigma Aldrich. Supplemented William’s Medium E (SWM) was prepared by adding HEPES (2 mmol/L) and *L*-glutamine (20 mmol/L) to William’s medium E. The solution was stored at 4 °C until use in incubation experiments. Thawing medium, 0.4% trypan blue, and ten-donor-pooled cryopreserved human hepatocytes were supplied by Lonza (Basel, Switzerland). Human MOR, DOR, and KOR membrane and the GTP G_i_ Binding assay kit were purchased from Revvity (Milan, Italy). The kit included the following reagent and stock solutions: GTP Eu Cryptate reagent, GTP d_2_ antibody, GDP, magnesium chloride (MgCl_2_) GTPγS, G_i_ protein control and Stimulation Buffer.

### In silico metabolite prediction

The molecular structure of dipyanone was represented using SMILES (Simplified Molecular Input Line Entry System), a line notation system that encodes chemical structures as compact text strings. These SMILES strings, generated using ChemSketch (Advanced Chemistry Development, Inc.; v. 2020.1.2), were then utilised in GLORYx, an open-access software collaboratively developed by the University of Vienna, Austria and the University of Hamburg, Germany (Carlier et al. 2022). GLORYx was employed to predict phase I and phase II human metabolites of dipyanone. The software assigns a prediction score to each metabolite, indicating its likelihood of formation. Metabolites with a prediction score of 25% or higher were selected for further analysis. To simulate additional metabolic transformations, these selected metabolites were reprocessed through GLORYx, generating “second-generation metabolites”. The final score of each second-generation metabolite was calculated by multiplying its score with that of its corresponding first-generation metabolite. Again, only those with a final score of 25% or higher were retained. The selected metabolites were added to the LC-HRMS/MS inclusion list, and their corresponding metabolic transformations were included in the list of predicted transformations for data mining. This two-step prediction process aimed to more comprehensively model the potential metabolic pathways of dipyanone in the human body, accounting for both immediate and subsequent metabolic transformations. This approach aided in identifying a wider range of potential metabolites that might be formed during drug metabolism, which is crucial for understanding the drug’s behaviour and potential effects in the body.

### Hepatocyte incubations

Dipyanone incubations with human hepatocytes were carried out as previously described (Carlier et al. 2022; Taoussi et al. 2024). Briefly, the hepatocytes were thawed in 50 mL of thawing medium at 37 °C. After centrifugation (100 g, 5 min), the supernatant was discarded and the pellet was resuspended in 50 mL of SWM at 37 °C. Following a second centrifugation (100 g, 5 min), the supernatant was removed, and the pellet was resuspended in 2 mL of SWM at 37 °C. Cell viability was determined using the trypan blue exclusion method, and the SWM volume was adjusted to reach a concentration of 2 × 10^6^ cells/mL. In sterile 24-well culture plates, 250 µL hepatocyte suspension was gently mixed with 250 µL of dipyanone (20 µmol/L in SWM). The plates were incubated at 37 °C, and reactions were stopped after 0 or 3 h with 500 µL ice-cold acetonitrile, followed by centrifugation (15,000 g, 10 min). Negative (without SWM, hepatocytes, or dipyanone) and positive controls (diclofenac incubation) were incubated for 0 and 3 h under the same conditions to exclude nonspecific reactions and ensure proper metabolic activity. Incubates were stored at – 80° until analysis.

### Authentic samples

Biological samples from two dipyanone-positive forensic cases were analysed to confirm the in vitro metabolite predictions.

In case #1, postmortem femoral blood and urine were collected at the autopsy. Dipyanone concentrations in blood and urine were 720 and > 1000 ng/mL, respectively. No other substances of toxicological interest were detected.

In case #2, dipyanone concentrations in autoptic heart blood and urine were 80 and 5,500 ng/mL, respectively. Other substances of toxicological interest that were detected in blood included: 2-fluoromethamphetamine (96 ng/mL), 2-fluoroamphetamine (24 ng/mL), deschloroketamine (1.0 ng/ml), 2-fluoro-deschloroketamine (< 1.0 ng/mL), deschloro-*N*-ethylketamine (46 ng/mL), mitragynine (160 ng/mL), and 7-hydroxymitragynine (4.7 ng/mL). In urine: 2-fluoromethamphetamine (not quantified), deschloroketamine (23 ng/mL), 2-fluoro-deschloroketamine (> 50 ng/mL), deschloro-*N*-ethylketamine (> 50 ng/mL), 2-fluoroamphetamine (not quantified), mitragynine (> 200 ng/mL), and 7-hydroxymitragynine (> 200 ng/mL).

### Sample preparation for metabolite identification

#### Incubates

A 100 µL volume of incubate was mixed with 100 µL of acetonitrile and centrifuged for 10 min, 15,000 g at room temperature for protein precipitation (acetonitrile:SWM, 3:1, v/v). The supernatant was dried under a nitrogen stream at 37 °C. The residue was reconstituted in 100 µL of a mixture containing 95% mobile phase A (0.1% formic acid in water) and 5% mobile phase B (0.1% formic acid in acetonitrile). Again, the solution was centrifuged (10 min, 15,000 g) at room temperature. The resulting supernatant was transferred into vials with a glass insert, and 10 µL was injected into the chromatographic system for analysis.

#### Urine samples

Samples were thawed at room temperature. A 100 µL aliquot was mixed with 200 µL of acetonitrile and centrifuged for 10 min at 15,000 g at room temperature. The supernatants were evaporated to dryness under nitrogen at 37 °C. The residues were reconstituted in 100 µL of a mixture containing 95% mobile phase A and 5% mobile phase B (v/v). After centrifugation under the same conditions, the supernatants were transferred to autosampler vials with glass inserts. A 10 µL volume was injected into the chromatographic system for analysis.

To investigate glucuronic acid conjugation, 100 µL of urine was mixed with 10 µL of 10 mol/L ammonium acetate (pH 5.0) and 100 µL of β-glucuronidase (5000 units), then incubated at 37 °C for 90 min. Subsequently, 400 µL of ice-cold acetonitrile was added to the mixtures, which were centrifuged for 10 min at 15,000 g at room temperature. The supernatants were evaporated to dryness under nitrogen at 37 °C and reconstituted in 100 µL of a mixture containing 95% mobile phase A and 5% mobile phase B (v/v). After centrifugation under the same conditions, the supernatants were transferred to autosampler vials with glass inserts. A 10 µL volume was injected into the chromatographic system for analysis.

### Instrumental conditions for metabolite identification

The analyses were conducted by liquid chromatography-high-resolution tandem mass spectrometry (LC-HRMS/MS) with a DIONEX UltiMate 3000 liquid chromatographer coupled to a Q Exactive quadrupole-Orbitrap hybrid high-resolution mass spectrometer equipped with a heated electrospray ionisation (HESI) source from Thermo Scientific (Waltham, Massachusetts, USA).

### Liquid chromatography conditions

The compounds were separated using a Kinetex Biphenyl column (150 × 2.1 mm, 2 μm) from Phenomenex (Torrance, California, USA). The separation was achieved using mobile phases A and B at a flow rate of 0.4 mL/min. The gradient elution program was as follows: 2% B was held for 2 min, then increased to 25% B over 12 min, followed by a rapid increase to 95% B within 2 min, and held for 4 min. Subsequently, the initial conditions were restored within 0.1 min and maintained for 3.9 min. The total chromatographic run time was 24 min. Throughout the analysis, the column oven temperature was maintained at 37 ± 1 °C, while the autosampler temperature was set to 10 ± 1 C.

### Mass spectrometry conditions

All samples were analysed in both positive- and negative-ion modes, requiring two separate injections while maintaining the same HESI conditions. These conditions were as follows: spray voltage, ± 3.5 kV; sheath gas and auxiliary flow rates, 50 a.u. and 10 a.u., respectively; capillary temperature and auxiliary gas heater temperature, 300 °C; and S-lens radio frequency level, 50 a.u.; Notably, the sweep gas flow rate was not utilised. Prior to each analytical session, mass calibration was performed using certified calibration solutions in both positive and negative ion modes. To enhance accuracy, a lock mass list was compiled for positive- (*m/z* 279.0933, 144.9821, 146.9803) and negative ion modes (*m/z* 265.1479, 162.9824, 248.9604).

The mass spectrometer acquired from 1 to 20 min of the chromatographic run in full-scan HRMS (FullMS)/data-dependent MS/MS (ddMS^2^) mode. The FullMS settings were as follows: range, *m/z* 100 to 650; resolution at full width at half maximum at *m/z* 200, 70,000; automatic gain control target, 1 × 10^6^; and maximum injection time, 200 ms. For ddMS^2^, the settings were: automatic gain control target, 2 × 10^5^; maximum injection time, 64 ms; isolation window, *m/z* 1.2; resolution, 17,500; and stepped normalised collision energy, 40, 70, and 90 a.u. A maximum of five ddMS^2^ scans were triggered for each FullMS scan, with a minimum intensity of 10^4^ and dynamic exclusion of 2.0 s..

The data-dependent acquisition relied on an inclusion list of putative metabolites (Supplementary Table S1) based on in silico predictions (Supplementary Table S2) and extrapolation from the metabolism of structural analogues (Ferrari et al. 2004; Manier et al. 2024).

Additionally, ions not included in the inclusion list could also trigger ddMS^2^ scans, albeit at a lower priority (using the “pick others if idle” option). Furthermore, an exclusion list was compiled based on background noise, as evaluated during the injection of blank control samples (A:B 95:5 v/v).

### Data mining for metabolite identification

LC-HRMS/MS data were processed with Thermo Scientific Compound Discoverer in a single analysis. Following a previously described workflow (Berardinelli et al. 2024; Taoussi et al. 2024), the detected ions were compared to a list of theoretical metabolites. This list was generated according to the settings displayed in Supplementary Table S3, with an intensity threshold of 5 × 10^3^ and an HRMS mass tolerance of 5 ppm. Additionally, the HRMS/MS spectra and theoretical elemental compositions of the ions were compared to mzCloud (Drugs of Abuse/ Illegal Drugs database), ChemSpider (Cayman Chemical, DrugBank), and HighResNPS online databases. For these comparisons, an intensity threshold of 10^5^; an HRMS mass tolerance of 5 ppm, and an HRMS/MS mass tolerance of 10 ppm were applied. For final identification, the intensity threshold was set at 1% of the signal of the most intense metabolite in the corresponding sample.

### HTRF-based GTP G_i_ binding assay

MOR, KOR, and DOR activation were evaluated through a HTRF®-based GTP G_i_ binding assay. In this assay, a drug is incubated with a membrane preparation from cells that express recombinant or endogenous receptors, containing G protein-coupled receptors (GPCR), specifically MOR, KOR, or DOR. The activation of the GPCR through agonist binding leads to the replacement of the GDP nucleotide in the receptor G alpha subunit by a non-hydrolysable GTP coupled to a fluorescent europium cryptate donor (Eu-PGT). When a d_2_-labelled anti-Gα_i_ monoclonal antibody acceptor is in proximity to the donor, a fluorescence resonance energy transfer (FRET) signal is emitted. This signal, proportional to the Gα_i_ activation state, can be measured at a specific wavelength (Koval et al. 2010; Rozwandowicz-Jansen et al. 2010).

The total assay volume was 20 µL, containing a supplemented stimulation buffer with optimised GDP and magnesium chloride concentrations, dipyanone or a reference compound (MOR, fentanyl; KOR, U-50488; DOR, SNC-80), a detection reagent mix of equal volumes of europium cryptate and d_2_-labelled antibody, and human MOR, KOR, or DOR membrane preparation. Non-specific binding was evaluated using a non-hydrolysable GTPγS at a saturation concentration (25 µmol/L) to measure the assay background signal. A positive control was prepared using a recombinant Gα_i_ subunit to which both the Eu-GTP analogue and the d_2_-antibody bind, allowing control of the detection reagent. Membranes were incubated overnight at room temperature. Dipyanone and the reference compounds were dissolved in a mixture of dimethyl sulfoxide and supplemented stimulation buffer (90:10, v/v), with final concentrations ranging from 10^–5^ to10^−11^ mol/L. Each concentration was tested in duplicates, and experiments were performed in triplicates (*n* = 3). The FRET signal was measured using a Multilabel Plate reader (PerkinElmer), calculating the fluorescence ratio of 665 to 620 nm to remove photophysical interference (delay: 100 μs; total time window: 200 μs). All values were normalised to the maximal signal of the corresponding reference receptor agonist, set to 100%. Concentration–response curves were fitted using GraphPad Prism (v. 10.2.3) with a three-parameter fit to determine potency (EC_50_) and efficacy (*E*_max_).

### Sample preparation for dipyanone quantification

#### Blood samples

Liquid–liquid extraction was performed on blood samples using acetonitrile and ammonium formate. The process began by fortifying 100 µL of blood or serum with an internal standard solution (methadone-d9, 10 ng/mL). Subsequently, 100 µL of ammonium formate (10 M) and 1.0 mL of ice-cold acetonitrile ( – 20 °C) were added and mixed for 5 min using an overhead shaker. The samples were then centrifuged at 4000 rpm for 10 min. Following centrifugation, the organic phase was transferred to an autosampler vial. This phase was evaporated to dryness under a stream of nitrogen at 40 °C and reconstituted in 100 µL of mobile phase (C/D, 90/10, v/v). Mobile phase C consisted of a 2 mM ammonium formate buffer with additives (0.1% formic acid and 1% acetonitrile), while mobile phase D was acetonitrile-based with additives (2 mM ammonium formate buffer and 0.1% formic acid).

#### Urine samples

Urine samples underwent an enzymatic conjugate cleavage prior to liquid–liquid extraction. Initially, 100 µL of urine was fortified with the same internal standard solution used for blood samples. Then, 100 µL of phosphate buffer and 10 µL of glucuronidase-arylsulfatase solution were added and incubated at 45 °C for 60 min. The subsequent extraction followed a similar procedure to that of serum, with adjusted extractant volumes: 200 µL of ammonium formate (10 M) and 1.5 mL of ice-cold acetonitrile ( – 20 °C).

### Instrumental conditions for dipyanone quantification

For the quantification of dipyanone, a semi-quantitative toxicological method validated for serum was used (Giorgetti et al. 2024). Briefly, analytical procedures were conducted using a QTRAP 5500 mass spectrometer from Sciex (Darmstadt, Germany) equipped with a Shimadzu Nexera X2 UHPLC-30AD system (Duisburg, Germany). Chromatographic separation was achieved on a Kinetex® F5 column (100 × 2.1 mm, 2.6 µm), along with a matching pre-column from Phenomenex (Aschaffenburg, Germany). The autosampler was set to maintain a temperature of 10 °C, and 10 µL of sample was injected into the system. The flow rate was set to 0.5 ml/min. Mobile phases consisted of an aqueous 2 mM ammonium formate buffer (with 0.1% formic acid and 1% acetonitrile) for phase C, and an acetonitrile-based eluent (with 2 mM ammonium formate buffer and 0.1% formic acid) for phase D. The gradient elution program was as follows: 0–1 min at 5% D, increase to 22.5% D from 1 to 4.5 min, increase to 32.5% D from 4.5 to 10.75 min, increase to 95% D from 10.75 to 13.5 min, hold at 95% D from 13.5 to 15.5 min, return to 5% D from 15.5 to 16 min, and hold at 5% D from 16 to 19.5 min. The total runtime was 19.5 min. Dipyanone was detected at 11.9 min (60 s detection window), while the internal standard methadone-d9 had a retention time of 10.6 min (90 s detection window). Mass spectrometry analysis was performed in positive electrospray ionization (ESI) mode with the following settings: ion spray voltage at 4500 V, curtain gas at 40 psi, collision gas set to medium, ion source gases at 60 psi (gas 1) and 70 psi (gas 2), and a source temperature of 500 °C. Data acquisition was performed using scheduled multiple reaction monitoring (sMRM). The sMRM parameters for dipyanone were as follows: Q1 m/z 336.0, transition 1 (Q3 m/z 265.0, collision energy (CE) 20 V), transition 2 (Q3 m/z 105.0, CE 35 V), transition 3 (Q3 m/z 219.0, CE 35 V). The sMRM parameters for methadone-d9 were: Q1 m/z 319.2, Q3 m/z 268.2, CE 20 V. All transitions were measured with an entrance potential of 10 V, a cell exit potential of 13 V and a declustering potential of 60 V, except for methadone-d9, where a declustering potential of 90 V was applied. Data analysis was performed using Analyst® software (version 1.5.1, Sciex, Darmstadt, Germany).

## Results

### In silico metabolite prediction

Metabolite prediction yielded five first-generation (pA_1_–pA_5_) and three second-generation (pA_X-1_–pA_X-2_) metabolites, ranked in decreasing order of prediction scores. For second-generation metabolites, pA_X_ represents the corresponding first-generation metabolite. These predictions are detailed in Supplementary Table S2. The predicted transformations were limited to reactions at the pyrrolidine ring, including *N*-oxidation, ring opening to *N*-butanal, hydroxylation, and subsequent *O*-glucuronidation or *O*-sulfation. All predicted metabolites were incorporated in the ddMS^2^ inclusion list for the LC-HRMS/MS analysis (see section “Mass spectrometry conditions”). Furthermore, all associated transformations were included in the list of potential reactions for the automated data mining (see section “Data mining for metabolite identification”).

### In vitro and in vivo dipyanone metabolite identification

**Fig. 1:**
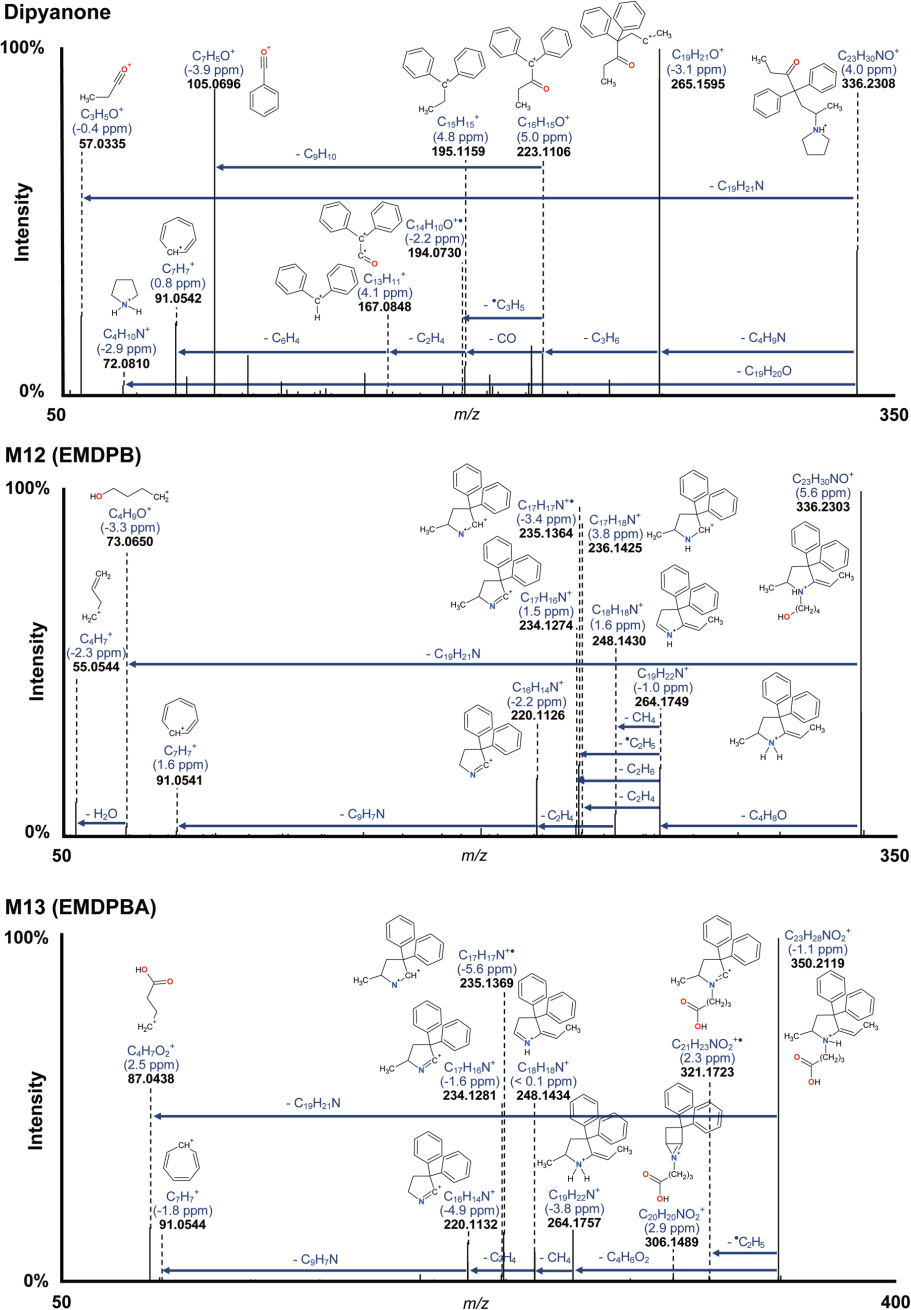
HRMS/MS spectra of dipyanone and major metabolites identified in human hepatocyte incubates and authentic postmortem samples, and suggested fragmentation patterns

Dipyanone ([M + H]^+^, mass-to-charge ratio m/z 336.2335, mass error – 4.0 ppm, C_23_H_30_NO^+^) eluted at 9.13 min. Notably, the molecule was not detected in negative ionisation mode. Consistent with the scientific literature, dipyanone yielded characteristic major fragments. These fragments were generated through two processes: first, a pyrrolidine loss yielding *m/z* 265.1578 (3.3 ppm, C_19_H_21_O^+^) and second, a subsequent propyl chain loss yielding *m/z* 223.1125 ( – 3.6 ppm, C_16_H_15_O^+^). Additionally, fragments at *m/z* 105.0337 ( – 2.4 ppm, C_7_H_5_O^+^) and *m/z* 91.0541 (1.2 ppm, C_7_H_7_^+^) representing the benzoyl and tropylium ions were detected. The dipyanone fragmentation spectrum is displayed in Fig. 1. Unless specified otherwise, the signals of dipyanone metabolites and their fragments are described in positive ionisation mode. Detailed fragmentation pathways are displayed in Supplementary Figs. 1–3.

### Metabolites identified in hepatocyte incubations

Following dipyanone incubation with human hepatocytes, eight metabolites were initially identified (M1, M4, M5, M10, M12-14, and M15, sorted by ascending retention time). Subsequently, minor urinary glucuronides (M2, M3, M6, M8, and M9) were retrospectively detected in hepatocytes, albeit at intensities below the identification threshold. However, M7 and M11 were not detected in hepatocyte incubations. After three hours of incubation, the dipyanone LC-HRMS/MS peak area decreased to approximately one-sixth of its original value compared to the controls. One of the two main metabolites, M12, was likely produced through ring opening and subsequent cyclisation, similar to the metabolism of methadone and its conversion to EDDP (for more details, see section “Pyrrolidine ring opening and cyclisation”). M12 and M13 constituted a substantial portion of the total metabolites’ signal, with M12 representing 50% and M13 accounting for 27%.

**Fig. 2:**
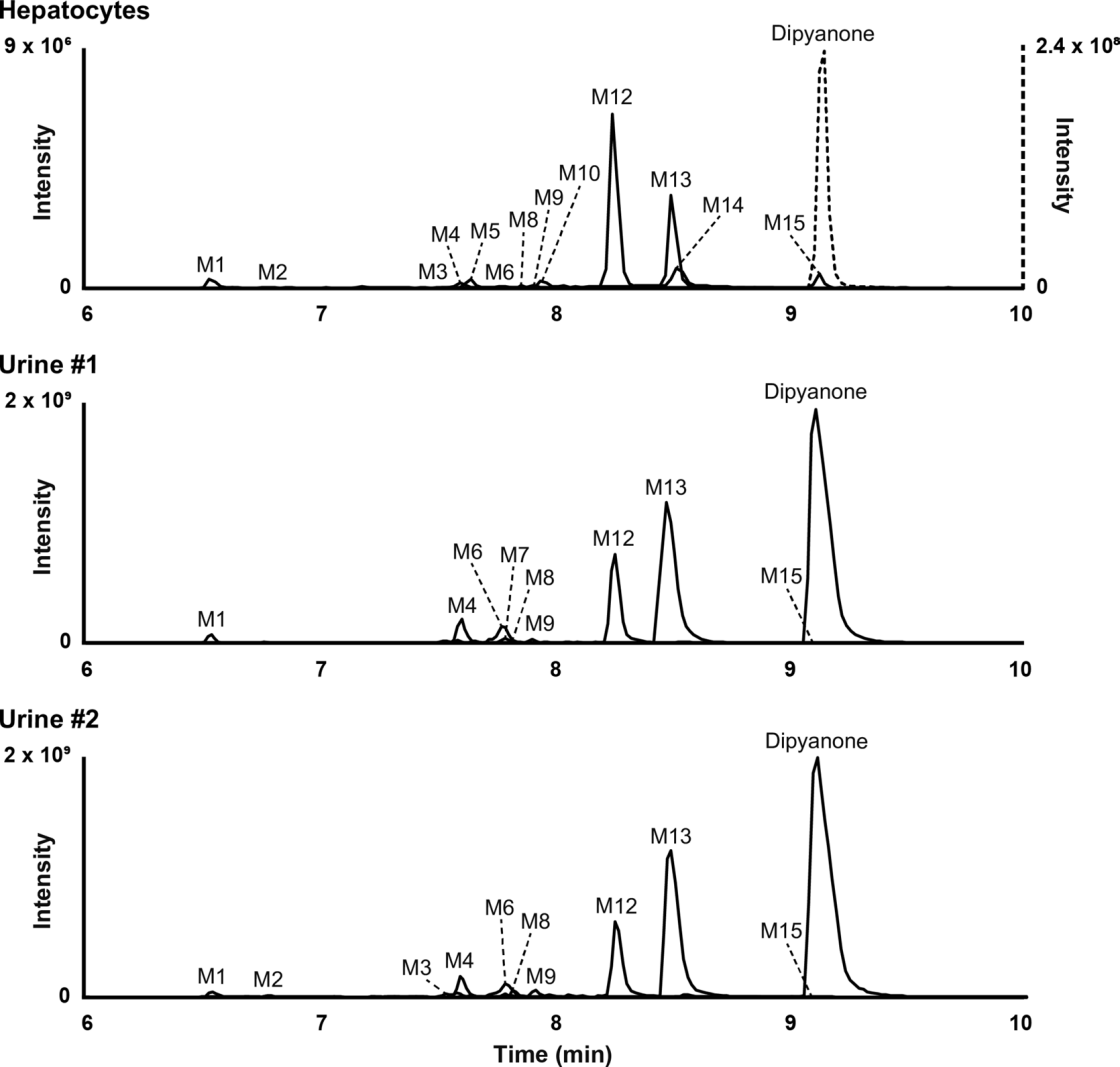
Extracted ion chromatograms of dipyanone and metabolites identified in human hepatocyte incubates and authentic postmortem samples. Mass tolerance, 5 ppm

Minor metabolites were generated through various transformations, including hydroxylation at the phenyl ring, the pyrrolidine ring, or the alkyl chain, as well as reduction and *O*-glucuronidation of M12. Table 1 reports the elemental compositions, retention times, accurate masses of the molecular ions, and LC-HRMS peak areas of dipyanone and its metabolites in positive ionisation mode after three hours of incubation with hepatocytes. Additionally, Fig. 2 displays the extracted ion chromatograms of dipyanone and its metabolites after the same incubation period.

**Table 1:** Metabolic transformation, elemental composition, retention time (RT), accurate mass of the molecular ion, deviation from theoretical accurate mass, and liquid chromatography-high-resolution mass spectrometry peak area of dipyanone and metabolites in positive ionisation mode after three hours of incubation with human hepatocytes and in postmortem samples

ID	Biotransformation	Elemental composition	RT, min	*m/z *(Δppm)	Hepatocytes	Urine #1		Urine #2	
					Peak area	Peak area	Peak area with β-gluc	Peak area	Peak area with β-gluc
M1	Reduction & Hydroxylation (phenyl) & Hydroxylation (phenyl)	C_23_H_31_NO_3_	6.55	370.2374 ( + 0.73)	9.6 × 10^5^	1.6 × 10^8^	1.8 × 10^8^	1.2 × 10^8^	1.1 × 10^8^
M2	Hydroxylation (phenyl) & *O*-Glucuronidation	C_29_H_37_NO_8_	6.78	528.2595 (0.58)	2.9 × 10^4^**	ND	ND	5.2 × 10^8^	ND
M3	Pyrr. op. to COOH & Cyclisation & *O*-Glucuronidation	C_30_H_31_N_4_O_4_	7.59	526.2441 (1.06)	1.0 × 10^4^**	ND	ND	5.7 × 10^7^	ND
M4	Pyrr. op. to butanol & Cyclisation & *O*-Glucuronidation	C_29_H_37_NO_7_	7.61	512.2645 (0.43)	5.0 × 10^5^	5.5 × 10^8^	ND	4.5 × 10^8^	ND
M5	Hydroxylation (alkyl)	C_23_H_29_NO_2_	7.67	352.2272 (0.27)	8.5 × 10^5^	ND	6.6 × 10^7^	ND	ND
M6	Pyrr. op. to COOH & Cyclisation &* O*-Glucuronidation	C_29_H_35_NO_8_	7.80	526.2444 (1.62)	3.4 × 10^4^**	1.1 × 10^8^	ND	1.7 × 10^8^	6.9 × 10^7^
M7	Pyrr. op. to butanol & Cyclisation & Hydroxylation (butanol)	C_23_H_29_NO_2_	7.81	352.2273 (0.55)	ND	6.1 × 10^7^	6.1 × 10^8^	ND	4.6 × 10^7^
M8	Hydroxylation (pyrr.) & *O*-Glucuronidation	C_29_H_37_NO_8_	7.83	528.2592 (0.01)	4.9 × 10^4^**	1.3 × 10^8^	ND	6.6 × 10^7^	ND
M9	Hydroxylation (pyrr.) & *O*-Glucuronidation	C_29_H_37_NO_8_	7.93	528.2592 (0.01)	6.8 × 10^4^**	1.2 × 10^8^	ND	6.9 × 10^7^	ND
M10	Hydroxylation (phenyl)	C_23_H_29_NO_2_	7.98	352.2273 (0.05)	7.3 × 10^5^	ND	1.8 × 10^8^	ND	6.8 × 10^7^
M11	Hydroxylation (phenyl)	C_23_H_29_NO_2_	8.11	352.2273 (0.05)	ND	ND	ND	ND	5.9 × 10^7^
M12	Pyrr. op. to butanol & Cyclisation	C_23_H_29_NO	8.25	336.2321 ( – 0.27)	**1.8 × 10**^**7**^	2.3 × 10^9^	3.4 × 10^9^	2.1 × 10^9^	1.7 × 10^9^
M13	Pyrr. op. to COOH & Cyclisation	C_23_H_27_NO_2_	8.50	350.2119 (1.29)	9.5 × 10^6^	**5.4 × 10**^**9**^	**5.7 × 10**^**9**^	**5.6 × 10**^**9**^	**3.3 × 10**^**9**^
M14	Hydroxylation (pyrr.)	C_23_H_29_NO_2_	8.57	352.2263 ( – 2.28)	3.8 × 10^6^	ND	3.7 × 10^8^	ND	3.5 × 10^8^
	**Dipyanone**	C_23_H_29_NO	9.12	336.2318	7.7 × 10^8^	1.1 × 10^10^	1.3 × 10^10^	1.3 × 10^10^	8.0 × 10^9^
M15	Hydroxylation (pyrr.)	C_23_H_29_NO_2_	9.15	352.2266 ( – 1.43)	1.2 × 10^6^	1.7 × 10^6^*	8.9 × 10^6^*	9.0 × 10^6^*	8.4 × 10^6^*

Mass tolerance, 5 ppm, *gluc* glucuronide, *NA* not applicable, *ND* not detected, *pyrr* *op* pyrrolidine opening, *Found in authentic samples after identification in incubates, **Found retrospectively in incubates after identification in authentic samples

### Metabolites identified in urine specimens

In case #1, eight metabolites were identified in the urine sample (M1, M4, M6-9, M12, and M13), with three additional metabolites (M5, M10, and M14) detected after glucuronide hydrolysis. Case #2 showed similar results, initially identifying nine metabolites (M1-4, M6, M8, M9, M12, and M13), and revealing four additional metabolites (M7, M10, M11, and M14) after glucuronide hydrolysis.

In both urine samples, consistent with the hepatocyte incubation results, M12 and M13 were predominant. However, their relative proportions differed from the hepatocyte incubations, representing 23–26% and 61%, respectively, of the total metabolites’ signal in urine. The dipyanone signal was approximately twice as intense as M13, the principal metabolite. Glucuronide hydrolysis did not substantially increase the signal of the main metabolites. Both urine samples contained traces of M15, a minor hydroxylated metabolite previously observed in vitro. However, its concentration was too low to meet the threshold for definitive identification. Table 1 compiles the in vivo results, and Fig. 2 displays the extracted ion chromatograms of dipyanone and its metabolites in both urine samples.

### Pyrrolidine ring opening and cyclisation

M13 eluted at 8.50 min with a molecular ion at *m/z* 350.2119 ( – 1.1 ppm, C_23_H_28_NO_2_^+^), suggesting the formation of an oxidised metabolite. The metabolite M12 eluted at 8.25 min at *m/z* 336.2303 (5.6 ppm, C_23_H_30_NO^+^), indicating the same elemental composition as dipyanone. However, M12 and M13 yielded entirely different fragments compared to dipyanone. While the HRMS/MS spectrum of dipyanone is dominated by fragments with a sum formula containing an oxygen atom, the spectra of M12 and M13 are mainly characterised by nitrogen-containing fragments. This observation suggests that the structures of M12 and M13 are fundamentally different from their parent dipyanone.

The analysis of M12 reveals a fragment at *m/z* 73.0650 ( – 3.3 ppm, C_4_H_9_O^+^) which likely results from a butanol chain cleavage. This finding suggests that the pyrrolidine ring was hydroxylated and subsequently opened. Similarly, M13 exhibits a fragment at *m/z* 87.0438 (2.5 ppm, C_4_H_7_O_2_^+^), indicating the loss of an alkyl chain with a terminal carboxy group. In both cases, the loss of the butyl chain leads to the same fragment (calculated *m/z* 264.1757, C_19_H_22_N^+^). This similarity implies that M12 and M13 share the same scaffold and only differ in their alkyl chains, with M12 having a hydroxyl group and M13 a carboxy group. Based on these assumptions, dipyanone could initially undergo enzymatic hydroxylation at the α position of the pyrrolidine ring. This specific process forms an unstable cyclic hemiaminal intermediate, whereas hydroxylation at the β position would not yield an unstable intermediate leading to ring opening. The hemiaminal can then follow two possible chemical pathways: firstly, it could undergo water loss, resulting in an enamine (not observed); alternatively, it could undergo ring opening, forming an alkyl chain with a terminal aldehyde group (as an intermediate further reduced to the corresponding alcohol or oxidised to the carboxylic acid). Although enzymatic dehydrogenation of the hemiaminal to a lactam is known in other metabolic processes (e.g. the transformation of nicotine to its metabolite cotinine), it was not observed in this study (Gorrod and Aislaitner 1994).

**Fig. 3:**
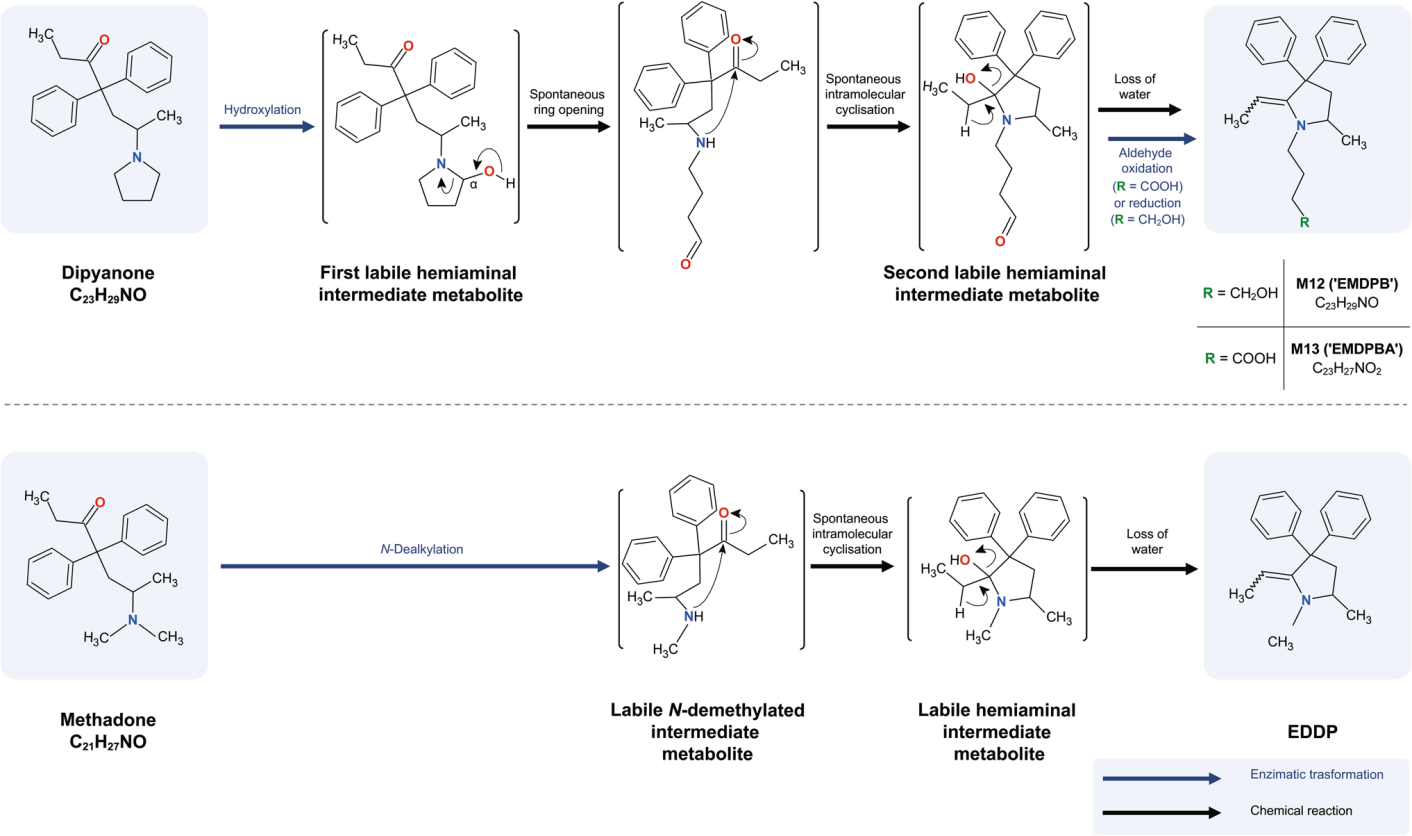
Comparison of EDDP formation to the proposed generation of metabolites M12 (“EMDPB”) and M13 (“EMDPBA”)

The transformation to M12 and M13 potentially continues in a manner analogous to the generation of methadone’s main metabolite 2-ethylidene-1,5-dimethyl-3,3-diphenylpyrrolidine (EDDP). EDDP results from *N*-demethylation followed by a nucleophilic attack of the nitrogen atom at the carbonyl carbon and a final water loss (Fig. 3) (Dilmaghanian et al. 2004; Overman and Ricca 1991; Pohland et al. 1971). Similarly, in the case of dipyanone metabolites after ring opening, the resulting secondary amine could also perform a nucleophilic attack at the carbonyl carbon adjacent to the two phenyl groups. This attack results in a five-membered ring and a second, intermediate hemiaminal. Once again, the labile hemiaminal has two options to stabilise, either by ring opening representing simply the reverse reaction or the loss of water finally yielding the scaffold of the two main metabolites of dipyanone (M12 and M13).

The intermediate metabolite (secondary amine carrying a butanal substituent) was not observed. Therefore, after the ring opening, the intermediate aldehyde is likely either reduced to a terminal alcohol or oxidised to a terminal carboxylic acid. These transformations would finally result in 4’-[2-**e**thylidene-5-**m**ethyl-3,3-**d**iphenyl**p**yrrolidin-1-yl]**b**utan-1’-ol (M12, EMDPB) and 4’-[2-**e**thylidene-5-**m**ethyl-3,3-**d**iphenyl**p**yrrolidin-1-yl]**b**utanoic **a**cid (M13, EMDPBA).

Figure 1 displays the fragmentation patterns of EMDPB and EMDPBA. The fragments are analogous to those yielded by 2-ethylidene-5-methyl-3,3-diphenylpyrrolidine (EMDP) (MzCloud, 2024), which is produced by EDDP *N*-demethylation and is another main metabolite of methadone. Although EMDP could potentially be a product of EMDPB or EMDPBA metabolisation after *N*-dealkylation, it was neither detected after hepatocyte incubation nor in the human samples.

### HTRF-based GTP G_i_ binding assay

**Fig. 4:**
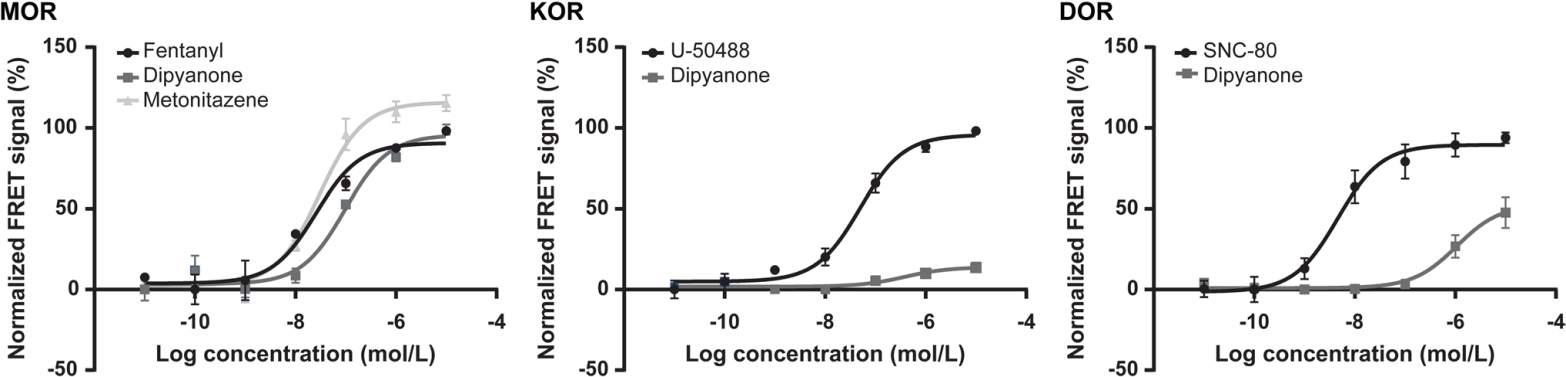
Dipyanone µ- (MOR), κ- (KOR), and δ- (DOR) opioid activation profiles (normalised to fentanyl, U-50488, and SNC-80 signal, respectively). Data are presented as mean ± SEM, *n* = 3; *FRET* fluorescence resonance energy transfer

The MOR, KOR, and DOR in vitro activation profiles represented by the normalised FRET signal intensity based on the concentration of dipyanone and controls in incubates are displayed in Fig. 4. Dipyanone EC_50_ and E_max_ at MOR, KOR, and DOR are reported in Table 2.

**Table 2:** Dipyanone in vitro µ- (MOR), κ- (KOR), and δ- (DOR) opioid receptor activation using a HTRF®GTP Gi binding assay, as represented by their potency (EC_50_) and efficacy (*E*_max_) relative to fentanyl (MOR), U-50488 (KOR), or SNC-80 (DOR)

Compound	MOR			KOR		DOR	
	EC_50_ [nM]	E_max_ [%] relative to fentanyl	E_max_ [%] relative to metonitazene	EC_50_ [nM]	E_max_ [%] relative to U-50488	EC_50_ [nM]	E_max_ [%] relative to SNC-80
Fentanyl	25.6 (10.1–70.2)	100 (98–119)	74 (68–88)	–	–	–	–
Metonitazene	33.6 (19.5–58.0)	130 (124–142)	100 (95–131)	–	–		–
U-50488	–	–	–	49.4 (29.6–80.1)	100 (98–113)		–
SNC-80	–	–	–	–	–	4.5 (2.1–9.7)	100 (89–108)
Dipyanone	96.8 (51.6–181.2)	106 (98–121)	81 (75–93)	380.4 (36.9–511.6)	13 (10–24)	1067 (516–1812)	56 (45–75)

Data are presented as mean with the 95% confidence intervals between parentheses; *n* = 3

## Discussion

### Dipyanone metabolism

**Fig. 5:**
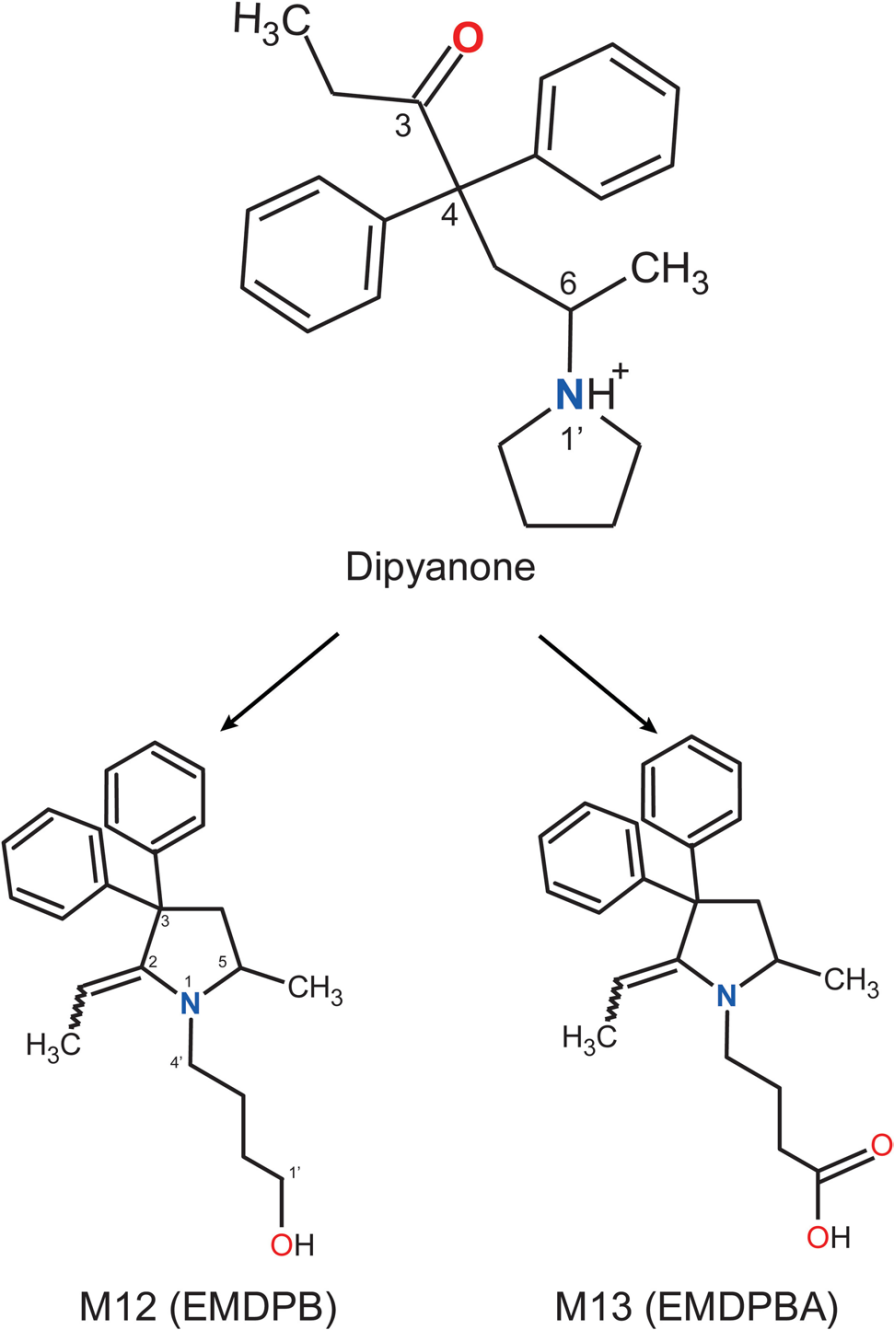
Dipyanone proposed metabolic fate in humans (major metabolites)

The metabolic fate of dipyanone is illustrated in Fig. 5. A strong correlation was observed between in vitro and in vivo samples, with the dominant metabolites detected in hepatocyte incubates also predominantly found in dipyanone-positive urine samples. Specifically, EMDPB (M12) and EMDPBA (M13) were identified as the major metabolites of dipyanone in both in vitro and in vivo studies, suggesting that these compounds may serve as specific biomarkers for dipyanone consumption. The predicted mechanism of formation for these metabolites involves several steps. Initially, dipyanone undergoes enzymatic hydroxylation, resulting in a labile hemiaminal. This unstable intermediate can undergo ring opening, leading to the formation of an alkyl chain with an aldehyde function. Subsequently, this aldehyde can be enzymatically transformed into either an alcohol or a carboxylic acid. Finally, an intramolecular ring closure and water loss yields the final main metabolites M12 and M13.

Similar metabolic reactions comprising ring opening have been observed in compounds containing piperazine and morpholine rings. For instance, the antibiotic linezolid, which bears a morpholine ring, undergoes a similar ring-opening mechanism via hemiaminal generation, leading to the formation of the respective alcohol and carboxylic acid metabolites (Pohland et al. 1971). Similarly, daridorexant, a medication used to treat insomnia, features a pyrrolidine ring which is opened after enzymatic hydroxylation to the hemiaminal. Comparable to M12 and M13, the resulting alkyl chain bearing an aldehyde group intramolecularly reacts via nucleophilic attack and subsequent closure to a six-membered ring (Treiber et al. 2023). In contrast, the major metabolite of nicotine does not undergo pyrrolidine ring opening. Instead, the α position is oxidised by aldehyde oxidase to the respective lactam via an immonium ion intermediate. This reaction was not observed for dipyanone, possibly due to the ring opening with the subsequent closure of a new ring followed by water loss being thermodynamically and/or kinetically more favourable (Pohland et al. 1971).

Regarding signal intensity, dipyanone was approximately four times more abundant than EMDPB and twice as abundant as EMDPBA in both urine specimens. Notably, enzymatic hydrolysis of urine specimens is not necessary to increase EMDPB or EMDPBA signal intensity. A study by Bernard et *al.* demonstrated in a large cohort that methadone urinary excretion is dependent on urine pH levels, while EDDP excretion is not (Bernard et al. 2007).In their study, the concentrations of methadone and EDDP were similar at a pH lower than 5.5, but the concentration ratio of methadone to EDDP decreased as the pH increased. Further investigations are necessary to determine whether a similar phenomenon occurs with dipyanone versus EMDPB and EMDPBA.

Methadone *N*-demethylation to EDDP has been shown to be primarily mediated by the cytochrome P450 (CYP) 2B6 isoenzym (Kreutzwiser and Tawfic 2020). The expression of CYP2B6 is highly variable between and within individuals due to various factors, including genetic polymorphisms, but also non-genetic factors like inducibility and inhibition by xenobiotics (Zanger and Klein 2013). If the same enzyme was responsible for dipyanone’s metabolic conversion to EMDPB and/or EMDPBA, the risk of overdose would possibly increase upon CYP2B6 inhibition. Moreover, the urinary concentration ratios of dipyanone to EMDPB and dipyanone to EMDPBA might greatly vary from one individual to another.

### In silico metabolite prediction

In silico metabolite predictions for dipyanone were largely inacurrate, failing to identify the two main metabolites observed in vitro and in vivo. This discrepancy can be attributed to dipyanone undergoing an uncommon metabolic reaction involving a nucleophilic attack leading to cyclization, which is not typically accounted for in standard prediction models. Current in silico methods, such as GLORYx, which uses machine learning-based sites of metabolism prediction and reaction rule sets, are likely not trained on datasets such reactions. This divergence between in silico and experimental results highlights the limitations of relying solely on computational methods for metabolite identification studies unless they are trained with complex training data sets. Until such sophisticated algorithms are available, thorough knowledge of unusual metabolic reactions and the underlying organic chemistry remains absolutely essential. Nevertheless, metabolite predictions can still serve as valuable tools for optimizing data mining efforts, as demonstrated in this study. To improve the accuracy of future prediction models, it is necessary to expand training datasets to include uncommon transformations and incorporating chemical reaction pathways that occur alongside biotransformations. This approach would better reflect the complex metabolic processes observed in real-world scenarios.

### Opioid receptors’ activation

Dipyanone’s pharmacological profile at MOR, DOR, and KOR was assessed using an HTRF-based GTP G_i_ binding assay.

Our findings indicate that dipyanone exhibits high potency at MOR (EC_50_, 96.8 nM), although approximately four and three times lower than that of fentanyl (EC_50_, 25.6 nM) and metonitazene (EC_50_, 33.6 nM), respectively. The efficacy of dipyanone was similar to that of fentanyl (E_max_, 106%, normalised to fentanyl), and lower than that of metonitazene (E_max_, 81%, normalised to metonitazene) (De Vrieze et al. 2024; Vandeputte et al., 2021a). The activity of fentanyl and metonitazene at MOR was previously studied using mini-G_i_ and β-arrestin recruitment assays, yielding values comparable to the present results (De Vrieze et al. 2024; Vandeputte et al., 2021a).

In these studies, the EC_50_ values of fentanyl and metonitazene ranged from 14.4 to 34.6 nM and 7.19 to 23.5 nM, respectively. Noteworthy, significant discrepancies may occur depending on the type and conditions of assays. For example, while the mini-G_i_ assay evaluates the receptor-G protein interaction itself, the GTP G_i_ binding focuses on the exchange of GDP for GTP on the G_i_ protein, and the β-arrestin assay occurs after receptor activation and phosphorylation by G-protein receptor kinases. It is important to consider that these analytical approaches are limited models that do not measure the ultimate functional outcomes and do not replicate various parameters such as the cell types, receptor expression levels, or physiological states, which may impact the drug activity. However, they provide quick results for clinical and forensic toxicologists to support clinical diagnoses, overdose management, and legal investigations. Using a β-arrestin2 recruitment assay, Vandeputte et al. measured a higher potency for both fentanyl (EC_50_, 9.35 nM) and dipyanone (EC_50_, 39.9 nM) at MOR when compared to the present results (Vandeputte et al. 2023); dipyanone was approximately four to five times more potent than fentanyl in both Vandeputte's study and our results. These results suggest that dipyanone is a potent MOR agonist and may potentially induce strong analgesia and euphoria, but it may also produce potentially fatal respiratory depression and dependence.

At DOR, dipyanone demonstrated the lowest activity (EC_50_, 1067 nM) and efficacy (*E*_max_, 56%) among the investigated compounds, which were substantially lower than for the reference compound SNC-80 (EC_50_, 4.5 nM). Results for the reference compound were consistent with the scientific literature (Charfi et al. 2014). Similarly, dipyanone exhibited low potency and the lowest efficacy at KOR among the investigated compounds when compared to the reference compound U-50488 (EC_50_, 380.4 nM; *E*_max_, 13%), whose results are consistent with the literature (Otte et al. 2022). These findings indicate that dipyanone activity at DOR and KOR is relatively low. This selective receptor activation profile may influence the pharmacological and toxicological properties of dipyanone which seems to be similar to that of methadone (Kristensen et al. 1994; Vandeputte et al. 2023).

Historical studies have provided valuable insights into dipyanone’s properties. In 1949, Bockmühl and Ehrhart demonstrated that methadone’s and dipyanone’s analgesic properties were approximately 5–10 times higher than those of pethidine (another methadone analogue sold under the brand name Demerol®) using the mouse tail pinch test (Bockmühl and Ehrhart 1949). Similar results were obtained by Janssen and Jageneau, who measured methadone and dipyanone median effective dose at 5.18 and 6.82 mg/kg, respectively, in a mouse hot plate assay (Janssen and Jageneau 2011). Other studies found a similar analgesic threshold for methadone and dipyanone at 1–2 mg/kg (Scott et al. 1947). Toxicity assessment in mice also showed similar LD_50_ values (~ 17 mg/kg) for both drugs (Scott et al. 1946, 1947). In accordance with the scientific literature, the present results indicate that dipyanone’s pharmacological profile is similar to that of methadone.

EMDPB and EMDPBA activities at the opioid receptors could not be evaluated in the present experiments due to the lack of commercially available pure standards. However, considering that EDDP does not substantially contribute to methadone’s therapeutic or psychoactive effects, it might be hypothesised that EMDPB and EMDPBA are also rather inactive. Nevertheless, further pharmacological evaluations would be necessary to verify this assumption.

### Dipyanone-positive cases

In case #1, dipyanone concentrations in blood and urine were 720 and > 1000 ng/mL, respectively, with no other substances of toxicological interest detected.

The blood concentration exceeds the previously reported value of 370 ng/mL in a postmortem case where a combination of central nervous system depressants, including dipyanone, was identified as the cause of death. In that case, dipyanone’s contribution to the death was not explicit (Vandeputte et al. 2023).

Dipyanone’s in vitro potency is comparable to that of methadone (Vandeputte et al. 2023), and potentially lethal blood concentrations for methadone ranged from 200 to 450 ng/mL according to Errico et al. (Errico et al. 2021) and exceed 400 ng/mL according to Schulz et al. (Schulz et al. 2020). Considering the present results and the data from the literature, the dipyanone blood concentration of 720 ng/mL measured in case #1 might have caused fatal respiratory depression.

In case #2, dipyanone concentrations in blood and urine were 80 and 5500 ng/mL, respectively. Several additional substances were also detected in blood, including, 2-fluoromethamphetamine, 2-fluoroamphetamine, deschloroketamine, 2-fluoro-deschloroketamine, deschloro-*N*-ethylketamine, mitragynine, and 7-hydroxymitragynine. Compared to classic amphetamines, designer amphetamines that are fluorine substituted at the aromatic ring are potent central nervous system stimulants with increased lipophilicity that might improve blood–brain barrier permeability (Rösner et al. 2005; Smart 2001). In contrast, dipyanone is a central nervous system depressant, and its relatively low blood concentration, in this case, suggests that it likely did not substantially contribute to the cause of death.

## Conclusions

Our study provides a comprehensive analysis of the metabolic fate and the opioid receptor activation profile of the methadone-like NSO dipyanone.

Through human hepatocyte incubations and the analysis of positive urine specimens, fifteen metabolites were identified. A strong correlation was observed between in vitro and in vivo metabolism. The main specific metabolic biomarkers of dipyanone consumption are produced by pyrrolidine ring opening and subsequent reduction/oxidation of the resulting aldehyde to the corresponding *N*-butanol or *N*-butanoic acid compound and cyclisation to EMDPB (M12) or EMDPBA (M13), similar to the metabolisation of methadone to EDDP. Further pharmacokinetic studies are warranted to identify the enzymes involved in dipyanone metabolisation and to determine the relevance of EMDPB and EMDPBA as biomarkers of consumption in clinical and forensic casework.

This study also highlights the current limitations of in silico metabolite prediction methods, particularly for compounds with complex metabolic pathways like dipyanone. While computational tools can assist in optimising data mining, they might fall short in accurately predicting metabolites formed through uncommon biotransformations. These findings underscore that thorough data mining of experimental data applying knowledge of unusual metabolic reactions and the underlying organic chemistry remain absolutely essential. Future advancements in predictive models should focus on incorporating a wider range of metabolic pathways, including rather exotic biotransformation reactions, to enhance their accuracy and applicability in drug metabolism research. In line with previously results (Vandeputte et al. 2023), dipyanone’s pharmacological profile of opioid receptor activation was similar to that of methadone, exhibiting high potency and efficacy at the MOR, and low potencies and efficacies at the DOR and KOR. The study would have benefited from having methadone as a control, to achieve a more accurate comparison under the same experimental conditions. Together with the reported death case, these findings suggest that dipyanone poses substantial health risks and presents abuse liability. To fully understand its pharmacology, additional pharmacodynamic studies should be conducted to evaluate the activity of dipyanone metabolites at the opioid receptors.

## Supplementary Information

Below is the link to the electronic supplementary material.

Supplementary file1 (PDF 424 KB)

Supplementary file2 (PDF 257 KB)

Supplementary file3 (PDF 191 KB)

Supplementary file4 (TIF 125804 KB)

Supplementary file5 (TIF 176256 KB)

Supplementary file6 (TIF 141621 KB)

## Data Availability

Derived data supporting the findings of this study are available from the corresponding author upon request.
