# Supplementary material for: Dipyanone, a new methadone-like synthetic opioid: In vitro and in vivo human metabolism and pharmacological profiling
Source: Arch Toxicol. 2025 Apr 29;99(6):2339–53. doi: 10.1007/s00204-025-04023-1 (PMC12185669; doi:10.1007/s00204-025-04023-1)
Supplement: Supplementary file 1 — Supplementary file1 (PDF 424 KB) [file 204_2025_4023_MOESM1_ESM.pdf]

Table S1. Inclusion list used during liquid chromatography-high-resolution tandem mass spectrometry (LC-HRMS/MS) for dipyanone metabolite identification.

Dipyanone

**Transformation**	**Elemental composition**	**$[M+H]^+$ *m/z***	**$[M-H]^-$ *m/z***
Parent (Dipyanone)	$C_{23}H_{29}NO$	336.2322	334.2176
+O	$C_{23}H_{29}NO_2$	352.2271	350.2126
+2O	$C_{23}H_{29}NO_3$	368.2220	366.2075
+2H	$C_{23}H_{31}NO$	338.2478	336.2333
-2H+O	$C_{23}H_{27}NO_2$	350.2115	348.1969
+2H+2O	$C_{23}H_{31}NO_3$	370.2377	368.2231
+2H+O	$C_{23}H_{31}NO_2$	354.2428	352.2282
-2H+2O	$C_{23}H_{27}NO_3$	366.2064	364.1918
-2H	$C_{23}H_{27}NO$	334.2165	332.2020
+6C+10H+6O	$C_{29}H_{39}NO_7$	514.2799	512.2654
+6C+8H+7O	$C_{29}H_{37}NO_8$	528.2592	526.2446
+2H+S+3O	$C_{23}H_{31}NO_4S$	418.2047	416.1901
+S+4O	$C_{23}H_{29}NO_5S$	432.1839	430.1694
-4C-6H	$C_{19}H_{23}NO$	282.1852	280.1707
-4C-8H-1O	$C_{19}H_{21}N$	364.1747	262.1601
-1H+2O	$C_{23}H_{28}NO_2+$	350.2115	349.2042
