# Supplementary material for: Dipyanone, a new methadone-like synthetic opioid: In vitro and in vivo human metabolism and pharmacological profiling
Source: Arch Toxicol. 2025 Apr 29;99(6):2339–53. doi: 10.1007/s00204-025-04023-1 (PMC12185669; doi:10.1007/s00204-025-04023-1)
Supplement: Supplementary file 2 — Supplementary file2 (PDF 257 KB) [file 204_2025_4023_MOESM2_ESM.pdf]

Table S2. Dipyanone putative metabolites predicted with GLORYx freeware and their prediction score (adjusted score for second-generation metabolites).

Dipyanone

ID	Transformation	Elemental composition	Score	Simplified molecular-input line-entry system (SMILES)
pA1	*N*-Oxidation	$C_{23}H_{29}NO_2$	29%	[O-][N+]3(C(CC(c1ccccc1)(c2ccccc2)C(=O)CC)C)CCCC3
pA2	Pyrrolidine opening to butanal	$C_{23}H_{29}NO_2$	29%	O=C(C(c1ccccc1)(c2ccccc2)CC(NCCCC=O)C)CC
pA3	Oxidation to γ-lactam	$C_{23}H_{27}NO_2$	29%	O=C3N(C(CC(C(=O)CC)(c1ccccc1)c2ccccc2)C)CCC3
pA4	Pyrrolidine hydroxylation (3')	$C_{23}H_{29}NO_2$	29%	O=C(C(c1ccccc1)(c2ccccc2)CC(N3CCC(O)C3)C)CC
pA4-1	+ *O*-Sulfation (hydroxyl)	$C_{23}H_{29}NO_5S$	29%	O=S(=O)(O)OC3CCN(C(CC(C(=O)CC)(c1ccccc1)c2ccccc2)C)C3
pA4-2	+ *O*-Glucuronidation (hydroxyl)	$C_{29}H_{37}NO_8$	28%	O=C(O)C4OC(OC3CN(C(C)CC(c1ccccc1)(c2ccccc2)C(=O)CC)CC3)C(O)C(O)C4O
pA5	Pyrrolidine hydroxylation (2')	$C_{23}H_{29}NO_2$	29%	O=C(C(c1ccccc1)(c2ccccc2)CC(N3CCCC3O)C)CC
pA5-1	+ O-Sulfation (hydroxyl)	$C_{23}H_{29}NO_5S$	28%	O=S(=O)(O)OC3N(C(CC(C(=O)CC)(c1ccccc1)c2ccccc2)C)CCC3
