# Supplementary material for: Dipyanone, a new methadone-like synthetic opioid: In vitro and in vivo human metabolism and pharmacological profiling
Source: Arch Toxicol. 2025 Apr 29;99(6):2339–53. doi: 10.1007/s00204-025-04023-1 (PMC12185669; doi:10.1007/s00204-025-04023-1)
Supplement: Supplementary file 3 — Supplementary file3 (PDF 191 KB) [file 204_2025_4023_MOESM3_ESM.pdf]

Table S3. Compound Discoverer processing settings for generating dipyanone putative metabolites.

**Dipyanone**	
**Phase I reactions**	Cyclisation 1 (–H → +O)<br>Cyclisation 2 (–4C –8H –O → ø)<br>Dehydration (–2H –O → ø)<br>Desaturation (–2H → ø)<br>Dihydrodiol formation (ø → +2H +2O)<br>Hydration (ø → +2H +O)<br>Ketone formation (–O → +2H)<br>Oxidation (ø → +O)<br>Oxidative deamination to alcohol (–2H –N → +H +O)<br>Oxidative deamination to ketone (–3H –N → +O)<br>Reduction (ø → 2H)
**Phase II reactions**	Acetylation (–H → +2C +3H +O)<br>Cysteine conjugation (–H → +3C +6H +N +2O +S)<br>Cysteine-Glycine conjugation (–H → +5C +9H +2N +3O +S)<br>Glucuronide conjugation (–H → +6C +9H +6O)<br>GSH conjugation (–H → +10C +15H +3N +6O +S)<br>Methylation (–H → +C +3H)<br>Sulfation (–H → +H +3O +S)
**Max number of dealkylations**	3
**Max number of phase II reactions**	2
**Max number of all steps**	5
**Adducts**	$[M+H]^+$<br>$[M-H]^-$
